# Is dissociation predicting the efficacy of psychological therapies for PTSD? Results from a randomized controlled trial comparing Dialectical Behavior Therapy for PTSD (DBT-PTSD) and Cognitive Processing Therapy (CPT)

**DOI:** 10.1017/S0033291724003453

**Published:** 2025-02-24

**Authors:** Nikolaus Kleindienst, Regina Steil, Kathlen Priebe, Meike Müller-Engelmann, Petra Lindauer, Annegret Krause-Utz, Franziska Friedmann, Christian Schmahl, Frank Enning, Martin Bohus

**Affiliations:** 1 Department of Psychosomatic Medicine and Psychotherapy, Central Institute of Mental Health, Medical Faculty Mannheim, Heidelberg University, Heidelberg, Germany; 2 Institute of Psychology, Goethe University Frankfurt am Main, Frankfurt, Germany; 3 Department of Psychiatry and Psychotherapy, Charité-Universitätsmedizin Berlin, Berlin, Germany; 4 Faculty Human Sciences, Department Psychology, Medical School Hamburg, Hamburg, Germany; 5 Faculty of Economics and Media, Psychology School, Hochschule Fresenius University of Applied Sciences, Cologne, Germany; 6 Faculty of Clinical Psychology, Leiden University, Leiden, The Netherlands; 7 Humboldt-Universität zu Berlin, Berlin, Germany; 8 McLean Hospital, Harvard Medical School, Boston, MA, USA; 9 Department of Clinical Psychology and Psychotherapy, Ruhr-University Bochum, Bochum, Germany

**Keywords:** childhood abuse, dialectical behavior therapy, dissociation, posttraumatic stress disorder, psychotherapy

## Abstract

**Background:**

Neuropsychological evidence suggests that dissociation might disturb emotional learning, which is a fundamental mechanism of psychotherapy. However, a recent meta-analysis on the impact of dissociation on treatment outcomes in psychotherapy trials for posttraumatic stress disorder (PTSD) reported inconsistent results and concluded that further high-quality clinical trials are needed to test whether dissociation affects the efficacy of psychotherapies. We had two main aims: First, to test whether the efficacy of two evidence-based psychotherapies for individuals with trauma-related PTSD is affected by the level of pretreatment dissociation. Second, we investigated whether a significant reduction in dissociation at an early stage of treatment is beneficial for subsequent efficacy.

**Methods:**

The potential impact of dissociation on efficacy was studied in 193 women with PTSD related to childhood abuse who were randomized to dialectical behavior therapy for PTSD (DBT-PTSD) or cognitive processing therapy (CPT). Efficacy was operationalized as a change in the Clinician-Administered PTSD Scale (CAPS). Dissociation was assessed with the Dissociation Tension Scale (DSS). The analyses accounted for major confounders (in particular initial PTSD severity).

**Results:**

Two main findings emerged from this study. First, baseline dissociation was a negative predictor for treatment efficacy. Second, a significant drop in dissociation at the initial stages of treatment was beneficial for subsequent efficacy.

**Conclusions:**

Dissociation likely reduces the efficacy of trauma-focused therapies. Accordingly, successful reduction of dissociation at an early stage of treatment assists the efficacy of trauma-focused psychotherapies.

## Background

Meta-analyses confirm that psychological therapies such as trauma-focused cognitive behavioral therapy (TF-CBT) or eye movement desensitization and reprocessing (EMDR) are effective in treating posttraumatic stress disorder (PTSD) (Lewis, Roberts, Andrew, Starling, & Bisson, 2020; Mavranezouli et al., 2020). However, many people diagnosed with PTSD do not respond satisfactorily to existing treatments. This is particularly true for certain subgroups, such as complex PTSD (CPTSD) and PTSD related to childhood abuse (CA), who struggle with pronounced disturbances in self-organization, emotion regulation, and identity. For studies including predominantly women with CPTSD, the meta-analysis by Dorrepaal et al. (2014) reported a mean improvement rate of only 35%, compared to an improvement rate of 65% for other PTSD studies. For psychotherapy studies that predominantly included individuals with childhood-onset trauma, the meta-regression by Karatzias et al. (2019) reported substantially lower efficacy when compared with psychological therapies including a majority of individuals with adult-onset trauma. Consequently, there is a need to improve treatment options for individuals with complex and/or childhood trauma-related presentations of PTSD.

Because evidence-based psychotherapies for PTSD rely on several common mechnisms and strategies such as reorganization of memory functions, modification of cognitions and emotions, psychoeducation, and acquisition of skills (Schnyder et al., 2015), a promising approach to improving the effectiveness of these therapies is to identify barriers that counteract these mechanisms. According to psychophysiological studies (e.g., the delayed conditioning experiment by Ebner-Priemer et al., 2009), dissociation is a potential barrier to the desired modification of information processing and memory: In contrast to both control groups (that is individuals without psychiatric diagnosis and individuals with borderline personality disorder (BPD) and low levels of dissociation), highly dissociating individuals with BPD failed to acquire differential conditioning response as assessed by valence, arousal, and skin conductance (Ebner-Priemer et al., 2009). These findings were corroborated from another Pavlovian study by Mauchnik, Ebner-Priemer, Bohus, and Schmahl (2010) using the same outcome parameters indicative of emotional learning. The authors reported that deficiencies in distinguishing conditioned signals of danger vs safety were specific to highly dissociating individuals with BPD and co-occurring PTSD.

While these neuropsychological findings suggest that dissociation may interfere with the efficacy of psychotherapeutic treatments, treatment studies linking dissociation to outcome remain inconclusive. In the only meta-analysis of the impact of dissociation on the effectiveness of psychotherapy for PTSD (Hoeboer et al., 2020), the impact of dissociation was small and not statistically significant (r = 0.06 for trauma-focused studies and r = 0.02 for non-trauma-focused studies). The authors concluded ‘that this meta-analysis provides no evidence for the idea that dissociation specifically reduces the effectiveness of trauma-focused treatment’ (Hoeboer et al., 2020, p.6). However, this finding may not apply to individuals with CPTSD. As argued by Brand (2024), the mean dissociation score in the majority of studies included in the meta-analysis (Hoeboer et al., 2020) was rather low which may have contributed to the nonsignificant finding. This argument is in line with Jowett, Karatzias, Shevlin, and Hyland (2022), who emphasize that dissociation may be particularly relevant for those with a diagnosis of CPTSD. Furthermore, the meta-analysis by Hoeboer et al. (2020) is inconclusive because the impact of dissociation on the change in PTSD severity may be more complex than this meta-analysis suggests. Importantly, there are two opposing aspects to dissociation. On the one hand, as noted above, high levels of dissociation can make it more difficult to achieve improvement of PTSD severity because dissociation may interfere with fundamental mechanisms of change (e.g., modification of memories). On the other hand, the supposedly negative effect of dissociation may be compensated because high levels of dissociation are strongly related to high levels of pre-treatment PTSD-severity (Brand & Stadnik, 2013; Carlson, Dalenberg, & McDade-Montez, 2012), which in turn are positively related to improvement of PTSD-severity (e.g., Haagen, van Rijn, Knipscheer, van der Aa, & Kleber, 2018). Taken together, these opposing aspects of dissociation may overlap and cancel each other out. A way to disentangle these opposing effects of dissociation is to statistically control for one of these effects. Accordingly, it is conceivable that a negative effect of dissociation on improvement is only seen when controlled for PTSD severity (Barnett, van der Pols, & Dobson, 2005). These theoretical considerations are empirically supported by Kleindienst et al. (2016) who found a clinically relevant impact of dissociation when controlling for PTSD severity. Notably, models that examine the potential effects of dissociation while controlling for the baseline severity of the outcome variable are widely used to predict the outcome of psychological treatments for adjacent diagnoses including BPD (e.g., Kleindienst et al., 2011; Spitzer, Barnow, Freyberger, & Joergen Grabe, 2007). However, in PTSD research, the impact of dissociation on the outcome while accounting for baseline severity of PTSD is limited to the study mentioned above (Kleindienst et al., 2016) which was conducted for dialectical behavior therapy for PTSD (DBT-PTSD) in a small inpatient sample and requires replication.

Furthermore, studies investigating whether a significant improvement in dissociation at an early stage of psychotherapy is beneficial for the subsequent efficacy in terms or PTSD severity are lacking. Such a study would be of both theoretical and clinical interest. Theoretically, a significant change in a mechanism that inhibits the process of change should moderate the magnitude of subsequent change. Clinically, it would be important to find empirically based starting points for individualized therapy, particularly as identifying dissociation at an early stage of treatment and then addressing dissociation in the hope of improving efficacy is common practice, but not yet evidence-based.

In summary, the current best evidence is insufficient to clarify whether dissociation is a clinically important predictor in psychotherapeutic treatments for PTSD, particularly in treatments for CPTSD. There is a lack of studies that address this question using models that control for baseline PTSD severity, which may act as a major confounder and thus mask the effect of dissociation. Furthermore, it would be interesting to investigate whether the effect of dissociation might be more pronounced in some types of psychological treatment. Finally, PTSD studies investigating the effects of a significant drop in dissociation during an early stage of intervention are lacking.

## Objectives

Our study has several aims. On a confirmatory basis, we aim to clarify whether pretreatment levels of dissociation predict the efficacy of two evidence-based psychotherapies for individuals with PTSD, that is DBT-PTSD and cognitive processing therapy (CPT). We hypothesized that high levels of dissociation would negatively affect efficacy. Given the high between-study heterogeneity reported in the Hoeboer et al. (2020) meta-analysis, we further tested for a differential effect by assessing whether the treatment group moderated the effect of dissociation. Finally, we examined whether a significant reduction in dissociation from the first to the second assessment was beneficial in terms of subsequent efficacy.

## Methods

### Trial design, patients

The data originate from a multicenter RCT comparing the efficacy of DBT-PTSD and CPT in 193 women diagnosed with CA-related PTSD (Bohus et al., 2020). Here, we present a hypothesis-driven post hoc analysis focusing on the moderating impact of dissociation. General procedures including randomization and recruitment are described elsewhere (Bohus et al., 2020; Kleindienst et al., 2021). Briefly, cis-women aged 18–65 years diagnosed with CA-related PTSD and meeting at least 3 DSM-5 criteria for BPD, including criterion 6 (affective instability) were eligible. Exclusion criteria comprised a diagnosis of schizophrenia or bipolar-I disorder (lifetime), substance dependence (current), medical conditions contradicting exposure, pregnancy, and psychopathology that required immediate treatment in a different setting (e.g., BMI < 16.5), and an unstable living situation (e.g., continued victimization by the perpetrator). Further exclusion criteria were treatment with DBT-PTSD or CPT in the previous year, mental retardation, and a life-threatening suicide attempt in the previous 2 months. Individuals with nonsuicidal self-injury, high-risk behavior, or suicide attempts with a low likelihood of death were not excluded. After providing written informed consent, participants were randomized to either DBT-PTSD or CPT. Diagnosticians were blinded to group allocation (concealed assignment). For further details, see Bohus et al. (2019, 2020) and the German Clinical Trials Register (registration number DRKS00005578). The study was approved by the pertinent ethics committee and complied with the Declaration of Helsinki.

### Treatments

DBT-PTSD is a modular psychological treatment tailored for CA-related complex PTSD (Bohus et al., 2019). DBT-PTSD integrates several evidence-based therapeutic strategies, including DBT principles and skills, trauma-specific cognitive and exposure-based techniques, compassion-focused interventions, and behavior change techniques. DBT-PTSD has been found to be efficacious in treating women with a history of CA and complex presentations of PTSD (Bohus et al., 2013; Bohus et al., 2020; Kleindienst et al., 2021). CPT focuses on identifying, challenging, and restructuring dysfunctional trauma-related cognitions and subsequent emotions such as denial and guilt (Resick et al., 2008). In our study, sessions 1–4 include client history, problem behaviors, and emergency procedures (Bohus et al., 2019). Sessions 5–16 include the original core CPT sessions using the cognitive-only version described by Resick and Schnicke (1992). In subsequent sessions, the client writes a statement about why the worst event happened and how it has affected her life, as well as beliefs about safety, trust, control/power, self-esteem, and intimacy. Worksheets are introduced to help the client identify/modify dysfunctional trauma-related beliefs. Both treatments have been parallelized with respect to time, frequency, and dose of therapy. Treatment adherence has been assessed for both CPT and DBT-PTSD. These assessments were based on specifically developed scales and indicated ‘good’ adherence for both treatments (Bohus et al., 2020).

### Diagnostic procedure and assessments

Diagnosis of PTSD was made using the Clinician-Administered PTSD Scale (CAPS-5, Weathers et al., 2018; Dittmann et al., 2017). Axis-I disorders were diagnosed using the Structured Clinical Interview (SCID-I, First, Spitzer, Gibbon, & Williams, 1997). All clients diagnosed with PTSD on the CAPS also fulfilled the diagnosis of PTSD on the SCID-I. BPD diagnoses were assessed using the International Personality Disorder Examination (IPDE, Loranger et al., 1994). Diagnostic interviews were conducted by experienced clinical psychologists who were blinded to treatment allocation.

The predefined primary endpoint was the improvement in PTSD severity from pretreatment (T1) to posttreatment (T6) on the CAPS-5 total score (Δ_16_CAPS). The CAPS-5 was administered with respect to the currently most distressing event (index trauma, Priebe et al., 2018). Dissociation was assessed from both the mean duration and intensity scores assessed with the Dissociation Tension Scale (DSS, Stiglmayr et al., 2010). The DSS includes 21 items assessing psychological and somatoform dissociation referring to the last week. In the original version by Stiglmayr et al. (2010), durations of these items (e.g., ‘I had that feeling as if my body did not belong to me’) are rated on scales ranging from 0% (never) to 100% (constantly). For those items that have been present during the last week, patients are asked to additionally indicate the maximum intensity during that week. These items were rated on 10-point Likert scales ranging from 0 to 9 (very high). Psychometric investigation of the DSS (Stiglmayr et al., 2010) supported convergent, discriminant, and differential validities of the mean duration score, and a high internal consistency of the 21 items (Cronbach’s α = 0.92, Gutmann’s split half r = 0.92, Stiglmayr et al., 2010). Furthermore, this study favored a one-factorial solution. Accordingly, we used the mean duration score. Theoretically, peak intensity should be even more relevant – as peak intensity is typically observed in highly stressful situations (Ebner-Priemer et al., 2009) such as exposure during trauma-focused psychotherapies. Accordingly, we additionally used the intensity of dissociation.

As described elsewhere (Bohus et al., 2020), main assessments were at baseline (=T1), 3 months (=T2), 6 months (=T3), 9 months (=T4), 12 months (=T5, end of high-frequency phase), and 15 months (=T6, postassessment).

### Statistical analysis

Two general linear models (GLMs) with Type I (that is sequential) sum of squares were used for investigating the research questions. These GLMs allow for i) combining dimensional (e.g., dissociation scores) and nominal variables (e.g., treatment) and ii) sequential testing of hypotheses while controlling for confounding variables.

The first model was used to test the hypothesis that a long duration and a high peak intensity of dissociation are detrimental to improvement in PTSD severity and explored whether these putative effects of dissociation may differ across treatment groups. Improvements were calculated as pre-to-post differences in the ITT-based CAPS scores (Δ_16_CAPS = CAPS_pre_ - CAPS_post_). Predictor variables included duration and intensity scores of dissociation and an interaction of these scores with treatment (DBT-PTSD vs CPT) within a saturated model. Based on theoretical grounds, two additional independent variables were included: treatment (DBT-PTSD vs CPT) and baseline PTSD-severity (CAPS_pre_). Treatment was included for two reasons: (i) treatment was found to be significantly related to Δ_16_CAPS (Bohus et al., 2020), (ii) for a proper interpretation of the dissociation*treatment interactions, a saturated model including treatment as a main effect is required. CAPS_pre_ was included because CAPS_pre_ is linked to both the level of dissociation and Δ_16_CAPS and hence is required in the model to disentangle the opposing effects of dissociation described above. A detailed discussion on suitable modeling of Δ-scores in this situation has been provided by Barnett et al. (2005); a substantive discussion in the context of dissociation has been provided by Spitzer et al. (2007) and Kleindienst et al. (2011, 2016).

The second GLM was used for exploring whether a significant drop in dissociation at an early stage of treatment (that is from T1 to T2) is beneficial for subsequent efficacy (that is improvement in the PTSD severity from T2 to T6, Δ_26_CAPS). A significant drop in dissociation was defined as a reliable improvement in both duration and severity of dissociation according to the reliable change criterion by Jacobson and Truax (1991), that is a drop of at least 12.43 with respect to duration and 1.30 points with respect to severity. In analogy to the first model, the predictor was included both as a main effect and – within a saturated model – as an interaction term with the treatment. To specifically carve out the effect of a drop (not the level) in dissociation, control variables included the levels of both PTSD severity and dissociation. Furthermore, the improvement in PTSD severity from T1 to T2 (Δ_12_CAPS) has been included. We think it is important to include this additional control variable to avoid confounding a specific drop in dissociation with a generally successful initial treatment phase.

Given the relatively high level of complexity used in the modeling, we sought ways to create a more tangible representation of the results. Accordingly, we supplemented the main results of the first GLM with partial correlations (controlling for pretreatment PTSD severity), which were conducted and plotted separately for the two treatments (CPT and DBT-PTSD) and for the two modalities of dissociation assessment (duration and intensity).

All data were checked for significant deviations from normality, linearity, and homoscedasticity. If influential outliers have been detected by Cook’s D, that is if Cook’s D exceeds the criterion of 4/sqrt(n) as implemented as the default criterion in the software used (SAS™ 9.4), we supplemented the analysis based on all cases by a sensitivity analysis after removing the respective outliers. All patients who had been randomized according to protocol were included (n = 193) in the analyses (see Bohus et al., 2020). To minimize bias and loss of power, inferential statistics were based on intent-to-treat (ITT) analyses, which rely on methods that avoid both overfitting and bias (Enders, 2006). The exact procedure for handling missing data has been published elsewhere (Bohus et al., 2020). In short, we combined the efficiency of stochastic regression imputation (SRI) at the item level (when ≤10% of items were missing) with the advantages of multiple imputation at the scale level. Regression models used for imputation on the item level were based on all other items in the scale. Models underlying multiple imputations were based on scores preceding and following the score to be imputed, dropout status, and treatment group. P-values ≤0.05 (two-tailed) were considered statistically significant.

## Results

### Characterization of the sample

Of the 193 women who were randomized according to protocol, 98 were allocated to DBT-PTSD and 95 to CPT. Patient characteristics are provided in Table 1. The differences between CPT and DBT-PTSD were not statistically significant.

**Table 1.** Patient characteristics at study entry

	Entire sample	CPT	DBT-PTSD
Age in years, mean (SD)	36.3 (11.1)	35.5 (11.4)	37.0 (10.7)
Education (n)^a^			
No graduation or still at school	5.8% (11)	4.3% (4)	7.2% (7)
Lower secondary school (‘Hauptschule’)	15.8% (30)	15.1% (14)	16.5% (16)
Intermediate secondary school (‘Mittlere Reife’)	35.3% (67)	36.6% (34)	34.0% (33)
High school graduation (‘Abitur’)	39.5% (75)	40.9% (38)	38.1% (37)
Other	3.7% (7)	3.2% (3)	4.1% (4)
Number of Axis I disorders, current, mean (SD)	3.25 (1.43)	3.44 (1.53)	3.06 (1.31)
Number of Axis I disorders, lifetime, mean (SD)	4.21 (1.54)	4.35 (1.62)	4.07 (1.45)
Co-occurring BPD (n)	48.2% (93)	53.6% (50)	43.9% (43)
Suicide attempts, lifetime (n)^b^	57.5% (107)	52.1% (49)	63.0% (58)
Nonsuicidal self-injury (at least once during the last month) (n)^c^	39.1% (75)	37.2% (35)	40.8% (40)
Index trauma (n)			
Sexual abuse or sexual and physical abuse	74.6% (144)	72.6% (69)	76.5% (75)
Exclusively physical abuse	25.4% (49)	27.4% (26)	23.5% (23)
Repeated (versus single) abuse (n)^c^	90.6% (174)	92.6% (88)	88.7% (86)
Age at first abuse in years, mean (SD)	7.69 (4.21)	7.71 (4.16)	7.67 (4.28)
Duration of abuse in years, mean (SD)	6.90 (6.00)	7.44 (6.69)	6.36 (5.16)
Psychotropic medication at baseline (n)^c^			
Any psychotropic medication	69.3% (133)	69.2% (65)	69.4% (68)
Antidepressants	53.7% (103)	54.3% (51)	53.1% (52)
Neuroleptics	28.7% (55)	33.0% (31)	24.5% (24)
Mood stabilizers^d^	2.1% (4)	3.2% (3)	1.0% (1)
Benzodiazepines	7.3% (14)	7.5% (7)	7.1% (7)
Other psychotropic medication	9.9% (19)	12.8% (12)	7.1% (7)

Data are expressed as mean (SD) or percentages (number) of participants.

a Data regarding education were available for 190 participants.

b Data regarding suicide attempts (lifetime) were available for 186 participants.

c Data regarding nonsuicidal self-injury, repeated abuse, and psychotropic medication were available for 192 participants.

d For details, see Bohus et al. (2020).

A total of 32.1% (62/193) of the participants dropped out of the study (CPT: 39.0%, DBT-PTSD: 25.5%). Of those who dropped out, 10 participants were excluded from the study because they were hospitalized for at least 2 weeks which was a predefined reason for stopping the study early. Half of those who dropped out (31/62) dropped out during the first 3 months; the other half dropped out after month 3. The dropout rate was substantially higher in the n = 93 participants with a double diagnosis of PTSD plus BPD (41.94%) than in the n = 100 participants who had no co-occurring BPD (23.00%).

### PTSD severity and dissociation

On average, pretreatment PTSD severity as assessed by the CAPS score was 40.4 ± 9.94. The mean duration and intensity of dissociation as assessed by the DSS were 24.0 ± 15.85 and 2.97 ± 1.67, respectively. In both groups, PTSD severity declined from pretreatment to posttreatment (CPT: CAPS_pre_ = 40.96 ± 8.95, CAPS_post_ = 26.41 ± 16.04; DBT-PTSD: CAPS_pre_ = 39.93 ± 10.84, CAPS_post_ = 20.56 ± 15.81). Similarly, both the frequency and intensity of dissociative symptoms declined in either treatment groups (DSS, duration: CPT: DSS_dur,pre_ = 23.96 ± 14.81, DSS_dur,post_ = 20.87 ± 18.08; DBT-PTSD: DSS_dur,pre_ = 24.13 ± 16.88, DSS_dur,post_ = 14.04 ± 14.58; DSS, intensity: CPT: DSS_int,pre_ = 3.12 ± 1.62, DSS_int,post_ = 2.61 ± 1.88; DBT-PTSD: DSS_int,pre_ = 2.82 ± 1.70, DSS_int,post_ = 1.77 ± 1.70).

In the first model, improvements from pretreatment (T1) to posttreatment (T6) in PTSD severity, as measured by the change in CAPS score (Δ_16_CAPS), were significantly predicted from the pretreatment scores in the DSS when controlling for pretreatment PTSD severity (CAPS_pre_). As shown in Table 2, both the duration and the intensity of dissociation were negatively associated with improvement in the CAPS scores (*F_1,186_* = 4.15, p = 0.043, and *F_1,186_* = 5.65, *p* = 0.018, respectively). Remarkably, the intensity of dissociation predicted Δ_16_CAPS beyond the duration of dissociation. It is also noteworthy that – as expected – a high level of baseline PTSD severity (CAPS_pre_) was positively related to change in the CAPS (Δ_16_CAPS; *F_1,186_* = 8.37, *p* = 0.0043). Along with the information that both baseline scores of dissociation were positively correlated with baseline PTSD severity (*r*(DSS_int,pre_, CAPS_pre_) = 0.44, *p* < 0.0001, *r*(DSS_dur,pre_,CAPS_pre_) = 0.44, *p* < 0.0001), it is plausible that high levels of baseline PTSD severity have the potential to mask a negative effect of baseline dissociation on treatment efficacy unless taken into account in a multivariate model.

**Table 2.** Results of the general linear model predicting pre-to-post improvements (Δ_16_CAPS) from the pretreatment frequency and intensity of dissociation while controlling for major confounders

Overall model predicting Δ_16_CAPS: *F_6,186_* = 4.30, *p* = 0.0004			
	Type-I sum of squares	*F*-value	*p*-value
1) Treatment	1119.45	*F_1,186_* = 5.67	0.0182
2) CAPS_pre_	1651.53	*F_1,186_* = 8.37	0.0043
3) DSS_dur,pre_	819.22	*F_1,186_* = 4.15	0.0430
4) DSS_int,pre_	1115.85	*F_1,186_* = 5.65	0.0184
5) Treatment * DSS_dur,pre_	263.61	*F_1,186_* = 1.34	0.2493
6) Treatment * DSS_int,pre_	120.57	*F_1,186_* = 0.61	0.4355

**Figure 1.**
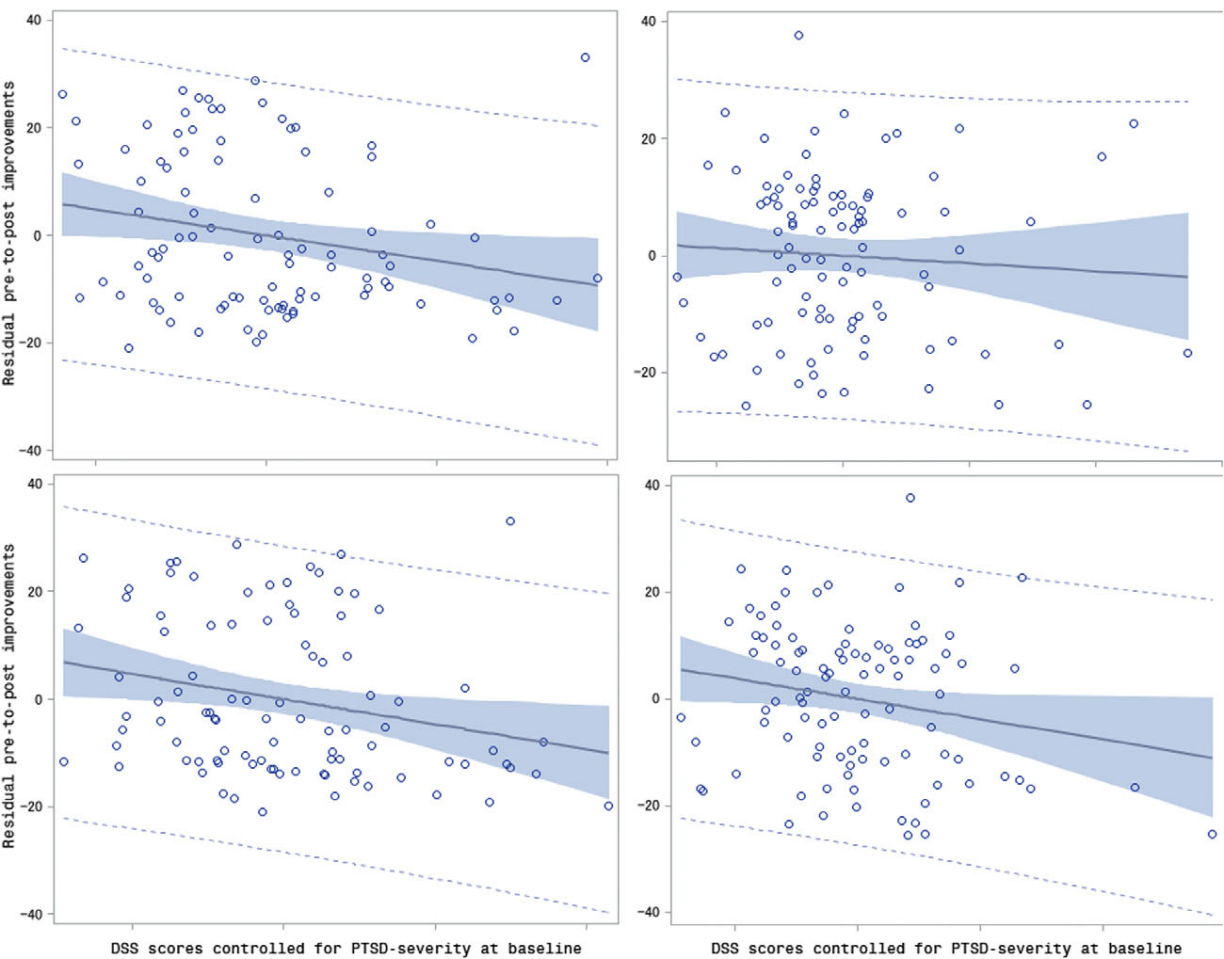
Scatterplot of pretreatment DSS scores while controlling for PTSD severity at baseline by residual pre-to-post improvements (Δ_16_CAPS). Linear fit (= solid line), 95% confidence area (= shaded area), and 95% prediction limits (dashed line). Left column: CPT, right column: DBT-PTSD, upper row: DSS duration, lower row: DSS intensity.

These findings were largely confirmed by partial correlations controlling for pretreatment PTSD severity conducted separately for the two treatments (CPT and DBT-PTSD). In the CPT group, Δ_16_CAPS was significantly associated with both the duration and intensity of the DSS (*r_part_*(Δ_16_CAPS, DSS_dur,pre_) = −0.23, *p* = 0.0277, and *r_part_*(Δ_16_CAPS, DSS_int,pre_) = −0.24, *p* = .0186, respectively). In the DBT-PTSD group, the partial correlation between Δ_16_CAPS and dissociation was significant only when the intensity score of the DSS was used (*r_part_*(Δ_16_CAPS, DSS_dur,pre_) = −0.07, *p* = .5009, and *r_part_*(Δ_16_CAPS, DSS_int,pre_) = −0.20, *p* = 0.0487, respectively). Overall, one highly influential outlier was detected (Cook’s D > 4/sqrt(n)). This outlier was found when calculating the partial correlation between Δ_16_CAPS and the duration of dissociation (*r_part_*(Δ_16_CAPS, DSS_dur,pre_). When a sensitivity analysis was performed excluding this outlier, the partial correlation remained significant and changed from −0.23 to −0.31 (*p* = .0021). The scatterplots underlying these partial correlations are depicted in Figure 1.

As shown in Table 2, the treatment*dissociation interaction terms were not statistically significant. Accordingly, the GLM provided no evidence for a differential effect of dissociation on the treatment group (CPT vs DBT-PTSD). This finding is consistent with the results from the regression analyses split for the two treatment groups (Figure 1).

Given the high dropout rates of 39.0% (CPT) and 25.5% (DBT-PTSD), the primary analysis based on ITT data was complemented by a sensitivity analysis based on data according to protocol (ATP). We carried out partial correlations (controlling for both group and baseline scores of the CAPS) between pre-to-post improvements (Δ_16_CAPS) and dissociation. Partial correlations were similar for ITT analyses (r_part_(Δ_16_CAPS, DSS_dur,pre_) = 0.2206, r_part_(Δ_16_CAPS, DSS_int,pre_) = 0.1488) and ATP analyses (r_part_(Δ_16_CAPS, DSS_dur,pre_) = 0.2297, r_part_(Δ_16_CAPS, DSS_int,pre_) = 0.1370).

To explore whether a significant reduction in dissociation from the first to the second assessment (that is during the first 3 months) is beneficial with respect to the subsequent efficacy during the next 12 months, a GLM modeling T2–T6 improvements in PTSD severity (Δ_26_CAPS) was calculated. As shown in Table 3, a drop in dissociation from T1 to T2 predicted further improvement beyond pretreatment PTSD severity, change in PTSD severity from T1 to T2, and baseline values of dissociation. Remarkably, the majority of participants who achieved a significant reduction in dissociation from T1 to T2 (Drop_12_DSS.RCI = 1) also achieved symptomatic remission of PTSD as assessed with the CAPS by the end of the study, although their initial dissociation scores were much higher than in those participants with no significant drop in dissociation from (duration: 49.3 ± 18.8 vs 24.7 ± 14.7, *t* = 4.46, p < 0.001; intensity: 4.12 ± 1.63 vs 2.86 ± 1.63, *t* = 3.05, p = 0.003).

**Table 3.** Results of the general linear model predicting improvements from T2 to T6 (Δ_26_CAPS) from the pretreatment frequency and intensity of dissociation while controlling for major confounders

Overall model predicting Δ_26_CAPS: *F_7,185_* = 5.60, *p* < 0.0001			
	Type-I sum of squares	*F*-value	*p*-value
1) Treatment	1125.19	*F_1,185_* = 6.58	0.0111
2) CAPS_pre_	0.19	*F_1,185_* = 0.00	0.9735
3) Δ_12_CAPS	2031.00	*F_1,185_* = 11.88	0.0007
4) DSS_dur,pre_	676.05	*F_1,185_* = 3.95	0.0482
5) DSS_int,pre_	609.68	*F_1,185_* = 3.57	0.0605
6) Drop_12_DSS.RCI	2081.84	*F_1,185_* = 12.18	0.0006
7) Treatment * Drop_12_DSS.RCI	173.73	*F_1,185_* = 1.02	0.3147

## Discussion

The aim of this study was to determine whether dissociation predicts a change in PTSD severity in individuals receiving a manualized psychotherapy for treating CA-related PTSD. As the participants in our study were randomized to either DBT-PTSD or CPT, we had the opportunity to further investigate whether dissociation might be a general predictor, or a treatment-specific predictor. In addition, the scheduled three-monthly assessments gave us the opportunity to explore whether a significant improvement early in treatment would be beneficial for later stages of treatment.

In our study, pretreatment dissociation was a negative predictor of change. This was true for both the duration and peak intensity of dissociation. Exploratory analyses indicated that a drop in both duration and intensity of dissociation during an early stage of treatment seemed to be beneficial for the remaining year of treatment.

These findings confirm and extend previous findings. The finding that pretreatment dissociation emerged as a negative predictor, when controlling for baseline PTSD severity, confirms a nuanced view of the role of dissociation in the treatment of trauma-related disorders. In line with previous models accounting for baseline severity (Kleindienst et al., 2011, 2016; Spitzer et al., 2007), these findings suggest that high levels of dissociation prevent psychological therapies from reaching their full potential. This finding does not contradict the finding by Hoeboer et al. (2020) who reported that the level of dissociation was not significantly correlated to the change in PTSD severity. Rather, our study contributes to a better interpretation of their findings. As confirmed in our study, the level of dissociation was positively correlated with baseline PTSD severity, which in turn was positively correlated to change in PTSD severity. Consequently, high levels of baseline dissociation are mostly present in individuals with high levels of baseline severity in PTSD, that is the group of individuals with the highest potential of improvement. However, as shown in analyses in which these interrelationships are incorporated into the modeling, the high potential for improvement (Barnett et al., 2005; Kleindienst et al., 2016) in highly symptomatic patients is attenuated by dissociation. The attenuation of efficacy related to dissociation does not argue against including highly dissociating individuals in currently used psychotherapies for PTSD. Despite overall high dissociation scores, there was a general improvement in PTSD severity, with no reliable worsening in any of the 193 participants (Bohus et al., 2020). The interpretation that baseline dissociation does not preclude successful psychotherapeutic treatment is further supported by our finding that individuals who started with high levels of dissociation, but showed a significant reduction in dissociation during the early stages of treatment, made particularly marked progress during the later stages of treatment, mostly achieving symptomatic remission.

When comparing our findings with published studies, it is important to remember that we investigated women with childhood trauma typically including severe abuse. The mean level of dissociation in our sample was relatively high. Accordingly, the disruptive role of dissociation during psychotherapy might be particularly pronounced in this type of individuals. Our finding of dissociation as a negative predictor of improvement may be partly related to the high level of dissociation observed in our sample.

The present study has both strengths and limitations. Strengths include the rigorous use of diagnostic procedures, interviews, and state-to-the-art therapies which were assessed for adherence during the study. Furthermore, outcome evaluation was based on the ITT sample, and the model used for evaluation was derived from the literature covering neuropsychological, clinical, and methodological findings. The main limitation relates to the observational design, which precludes conclusions about a causal influence of dissociation. Accordingly, while our aim was to improve trauma-focused psychotherapies by identifying mechanisms of change, our study is only a first step in this direction, which needs to be complemented by the evaluation of targeted manipulation of the putative mechanism of change. Further limitations relate to external validity, which is restricted by the inclusion and exclusion criteria used in our study. In particular, we exclusively included cis-women with an index trauma related to childhood sexual or physical abuse. As is typical for this type of trauma, the average levels of PTSD severity and dissociation were relatively high. Therefore, the results of our study should not be readily extrapolated to other populations such as men or individuals with PTSD from different trauma types. It is conceivable that the relation between dissociation and efficacy is less pronounced in less symptomatic samples. Similarly, we only looked at two specific trauma-focused therapies – CPT and DBT-PTSD – that were delivered in a face-to-face format. Although trauma-focused psychotherapies share important techniques and mechanisms (Schnyder et al., 2015), any generalization must take into account that the findings relate primarily to the two psychotherapies investigated. Furthermore, these psychotherapies have been applied in a regulated framework that served to test the efficacy of manualized therapies while excluding potentially confounding factors such as psychiatric hospitalizations. This strict procedure resulted in the early termination of several participants and resulted in high dropout rates. However, sensitivity analyses did not indicate that the results were dependent on the completer status. Still, the high dropout rates should be considered clinically relevant and should be further addressed. Finally, assessments of dissociation did not include a diagnosis of dissociative disorders. Accordingly, we did not establish the percentage of participants with diagnoses of dissociative disorders. Further research is required to establish the potential impact of dissociative disorders on treatment outcomes in the context of PTSD. While these limitations affect external validity, we do not believe that they affect our main findings. The finding that dissociation assessed at baseline negatively predicts improvement is also consistent with neuropsychological findings supporting the idea that dissociation attenuates learning and memory processes (Ebner-Priemer et al., 2009).

Our study suggests several future directions. First, our findings should be replicated in studies that include clients with other types of trauma (e.g., veterans) and with different psychological treatments (e.g., EMDR). Furthermore, potential benefits in clinical practice should be explored. Accordingly, we need rigorously designed trials assessing the extent to which outcomes can be improved by treating dissociation more vigorously than is usually the case. While we believe that common strategies such as teaching skills, mindfulness, and reducing vulnerabilities can reduce dissociation, further methods to detect and eventually address dissociation would be extremely helpful. One approach might be to assess dissociation during therapy sessions and provide contingent instruction and implementation of antidissociative strategies as part of trauma-focused psychotherapy.
